# Identification of Two Novel Pathogenic Variants of the *ATM* Gene in the Iranian-Azeri Turkish Ethnic Group by Applying Whole Exome Sequencing

**DOI:** 10.2174/0113892029268949231104165301

**Published:** 2023-12-28

**Authors:** Amir-Reza Dalal Amandi, Neda Jabbarpour, Shadi Shiva, Mortaza Bonyadi

**Affiliations:** 1 Animal Biology Department, Faculty of Natural Sciences, University of Tabriz, Tabriz, Iran;; 2 Pediatric Health Research Center, Tabriz University of Medical Sciences, Tabriz, Iran;; 3 Center of Excellence for Biodiversity, Faculty of Natural Sciences, University of Tabriz, Tabriz, Iran

**Keywords:** *ATM* gene, breast cancer, whole exome sequencing, c.2639-2A>T, c.8708delC, c.6067G>A, c.7788G>A

## Abstract

**Background:**

The *ATM* gene encodes a multifunctional kinase involved in important cellular functions, such as checkpoint signaling and apoptosis, in response to DNA damage. Bi-allelic pathogenic variants in this gene cause Ataxia Telangiectasia (AT), while carriers of *ATM* pathogenic variants are at increased risk of cancer depending on the pathogenicity of the variant they carry. Identifying pathogenic variants can aid in the management of the disease in carriers.

**Methods:**

Whole-exome sequencing (WES) was performed on three unrelated patients from the Iranian-Azeri Turkish ethnic group referred to a genetic center for analysis. WES was also conducted on 400 individuals from the same ethnic group to determine the frequencies of all *ATM* variants. Blood samples were collected from the patients and their family members for DNA extraction, and PCR-Sanger sequencing was performed to confirm the WES results.

**Results:**

The first proband with AT disease had two novel compound heterozygote variants (c.2639-2A>T, c.8708delC) in the *ATM* gene revealed by WES analysis, which was potentially/likely pathogenic. The second proband with bi-lateral breast cancer had a homozygous pathogenic variant (c.6067G>A) in the *ATM* gene identified by WES analysis. The third case with a family history of cancer had a heterozygous synonymous pathogenic variant (c.7788G>A) in the *ATM* gene found by WES analysis. Sanger sequencing confirmed the WES results, and bioinformatics analysis of the mutated ATM RNA and protein structure added evidence for the potential pathogenicity of the novel variants. WES analysis of the cohort revealed 38 different variants, including a variant (rs1800057, *ATM*:c.3161C>G, p.P1054R) associated with prostate cancer that had a higher frequency in our cohort.

**Conclusion:**

Genetic analysis of three unrelated families with ATM-related disorders discovered two novel pathogenic variants. A homozygous missense pathogenic variant was identified in a woman with bi-lateral breast cancer, and a synonymous but pathogenic variant was found in a family with a history of different cancers.

## 1 INTRODUCTION

The *ATM* gene is a relatively large gene, spanning 153,268 nucleotides and consisting of 66 exons [1-4]. It encodes a protein of 3056 amino acids that belongs to the PI3/PI4-kinase family and plays crucial roles in various cellular processes, including mitogenic signal transduction, chromosome stability, meiotic recombination, cell cycle control, and apoptosis [5-8]. The * ATM* protein serves as an essential kinase in the cell cycle, phosphorylating downstream proteins to regulate their functions. It interacts with a wide range of proteins, including checkpoint kinase CHK2, checkpoint proteins RAD17 and RAD9, tumor suppressor proteins P53 and BRCA1, and DNA repair protein NBS1 [2, 9-14]. Mutations in the *ATM* gene can lead to ataxia telangiectasia, breast cancer, and increased susceptibility to cancer development [15]. Ataxia telangiectasia is a disease with an autosomal recessive inheritance [15]. It is characterized by progressive cerebellar degeneration, premature aging, growth retardation, gonadal atrophy, immune deficiency, hypersensitivity to ionizing radiation, genomic instability, and cancer susceptibility [16-19]. Telangiectasia usually develops between the ages of 3 to 4 in parts of the face and corners of the eyes [20-22]. In addition to imbalanced movements, these patients may also have difficulties with speech, eye movements, and swallowing [23-26]. The frequency of the disease is 1 in 40000 to 100000 live births, depending on the ethnicity [27-29]. Studies have shown that germline mutations in *ATM* are associated with an increased risk of developing breast cancer, particularly in the context of familial breast cancer cases [30]. Furthermore, somatic alterations in the *ATM* gene have also been identified in sporadic breast tumors. These alterations include point mutations, deletions, and promoter hypermethylation, which can lead to the functional inactivation of * ATM* . Loss of * ATM* function compromises the ability of breast cells to repair DNA damage effectively, thereby promoting the accumulation of additional genetic alterations and increasing the risk of tumor initiation and progression [31]. The involvement of * ATM* in breast cancer extends beyond its role in DNA repair. * ATM* also influences other critical cellular processes, such as cell cycle control, apoptosis, and maintenance of telomere length. Dysregulation of these processes due to * ATM* dysfunction can contribute to breast cancer development and progression [32]. Understanding the contribution of *ATM* alterations to breast cancer pathogenesis is crucial for improved risk assessment, early detection, and the development of targeted therapies for patients with * ATM*-related breast cancer.

In this study, we applied WES to analyze three cases with ataxia telangiectasia, breast cancer, and susceptibility to the development of cancer diseases. Additionally, we investigated the frequencies of different variants in the *ATM* gene in our cohort from the Iranian-Azeri Turkish population using WES.

## 2 MATERIALS AND METHODS

A 12-year-old boy born to a non-consanguineous family originating from the Iranian-Azeri Turkish ethnic group was referred to the genetic lab for WES analysis. This boy was diagnosed with ataxia in early childhood. The other patient was a thirty-eight-year-old woman diagnosed with breast cancer. She was born to a family with a consanguineous marriage. The third case was a forty-year-old woman with a family history of multiple types of cancer. All three cases were analyzed by applying WES.

To study the frequencies of *ATM* variants in our population (Iranian-Azeri Turkish), we analyzed WES data from a cohort of 400 individuals. To accomplish this, we randomly selected 400 healthy individuals, ensuring an equal distribution of males and females, from the population and performed WES analysis on their samples.

According to the protocol, written consent was obtained from each patient or their guardian. Blood samples were taken from the * ATM* case and his parents in EDTA tubes. For the breast cancer case (the second case), the blood sample was taken only from the patient. Blood samples were also taken from the third case with a family history of multiple cancers and her parents. The DNA samples were extracted, the quality was checked, and finally, the DNA samples from the cases were subjected to WES as previously described [33, 34]. Briefly, WES experiments were performed using the Agilent SureSelect Human All Exon V7 Target Enrichment kit. Sequencings were performed from the enriched library on the Illumina NovaSeq 6000 platform, and sequence readings of 150bp were mapped to the human reference genome UCSC (University of California, Santa Cruz) (GRCh37/hg19 and GRCh38 / hg38) using Burrows-Wheeler Aligner (BWA) and CLC genomic desktop software (version 21). Low-quality reads and duplicates (Qbase < 20) were removed using Picard and Trimmomatics V0.39 tools, respectively. Sam-tools was used to sort and index bam files. The variants were then loaded into the wANNOVAR web tool [35] for annotation. The variants were prioritized according to the ACMG guideline. Filtration was first performed by removing variants located in the deep intronic sites, upstream, downstream, and ncRNA regions. In the next step, variants reported in different populations with a frequency greater than 0.05 were excluded, as they are known to be polymorphic. Finally, the list of genes that are attributed to ataxia was extracted from the OMIM [36] site and matched with the remaining variants. Candidate genes related to the ataxia disorder were selected and checked for the possibility of pathogenicity, likely pathogenicity, or VUS. To achieve this, different web-based tools were used, including VarSome [37], pathogenic variant taster [38], and Polyphen-2 [39]. In addition, the secondary structure of the RNA transcribed by the selected alleles was studied using web-based applications [40], and their effects on the structure and function of the encoded protein were evaluated using web-based tools [41]. Identified variants were evaluated with Alamut Pathogenic variant Interpretation Software (Interactive Biosoftware, Rouen, France) using different splice prediction algorithms, including SpliceSiteFinder, MaxEntScan NNSPLICE, and GeneSplicer. This tool allows the analysis of a single pathogenic variant to detect its impact on splicing active site scores (splice sites, branch points, and auxiliary sequences) [42-44].

After identifying the pathogenic variants in the probands, in order to confirm the results, Sanger sequencings were performed in the patients and other members of the family (in the case of availability) with ABI 3500 Genetic Analyzer (Applied Biosystems, Foster City, CA, United States). To achieve this purpose, primers from the interested regions were designed. All the DNA samples from the families (healthy and affected members) were subjected to PCR, followed by Sanger sequencing.

## 3 RESULTS

**Fig. (1):**
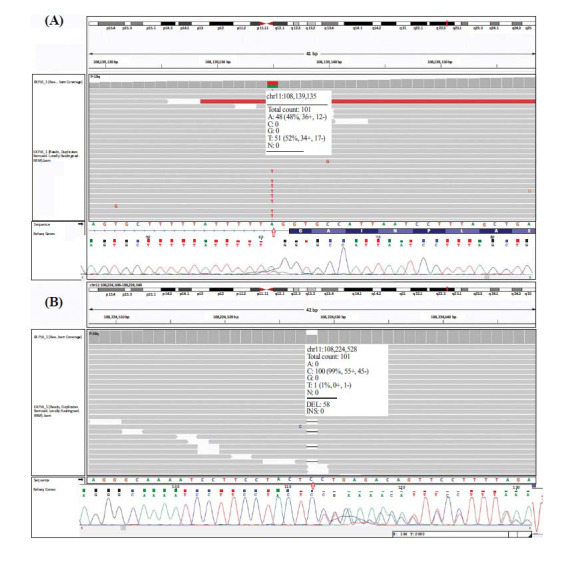
Whole exome sequencing and sanger sequencing results of the proband with * ATM* disease. In this patient, two variants were identified in the *ATM* gene: (c.2639-2A>T) and (c.8708delC). (**A**) The first variant (c.2639-2A>T), located on the splicing site, is depicted in the IGV figure of the BAM file of the proband (upper figure). This potentially pathogenic variant is shown in a heterozygous state in the sanger sequencing resulting from the proband (lower figure). (**B**) The second variant (c.8708delC), located on exon 60, is illustrated in the IGV figure of the BAM file of the proband (upper figure). The variant is shown in a heterozygous state in the sanger sequencing resulting from the proband (lower figure).

The first case was a 12-year-old boy with ataxia, who was referred to the genetic lab for genetic analysis. He has had progressive ataxia, oculomotor apraxia and a weakened immune system due to low IgG since his eight-year age. Telangiectasia was also seen in both eyes. Brain MRI had shown mild cerebellar white matter loss. No other malformation was observed in the brain MRI. WES analysis revealed two pathogenic variants of the *ATM* gene in this patient, one involving the splicing site on intron 17 (c.2639-2A>T) (Fig. **1A**) and the other potentially pathogenic variant was located on exon 60 (c.8708delC, p.P2903Lfs*34) resulting in the removal of nucleotide C, which causes a frame-shift pathogenic variant (Fig. **1B**). Both of the potentially/likely pathogenic variants were confirmed in the case and his parents by applying Sanger sequencing. The mother was confirmed to be a carrier for the c.2639-2A>T variant. The father was a carrier for the c.8708delC variant. His mother (the grandmother of our case) died of a brain tumor at the age of 30-year-old. Analysis of various splice prediction algorithms revealed that the c.2639-2A>T pathogenic variant disrupts the acceptor site of exon eighteen by 100 percent. The other variant, c.8708delC, results in a premature stop codon in exon sixty of the *ATM* gene. This premature stop codon is most likely detected by the nonsense-mediated mRNA decay (NMD) surveillance pathway and results in the degradation of the mRNA.

**Fig. (2):**
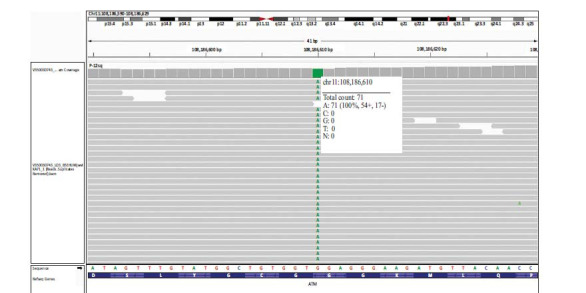
Whole exome sequencing results of the proband with bilateral breast cancer (case number two). The pathogenic variant in the *ATM* gene (c.6067G>A) is illustrated in the IGV figure of the BAM file of the proband (this pathogenic variant is in the homozygous stage). The total count of the read for this region is 71, all showing G to A pathogenic variant.

The second case was a 38-year-old woman with bilateral breast cancer. She was born to a consanguineous marriage and developed bilateral breast cancer at 35 years old. WES analysis identified a variant (c.6067G>A) in the *ATM* gene in a homozygous state (Fig. **2**). No other pathogenic variant was observed in any of the genes involved in cancer disease. There are conflicting interpretations of the pathogenicity of the c.6067G>A variant in Clinvar. However, according to computational analysis, this variant (rs11212587) is classified as pathogenic [37-41]. The analysis indicates that the variation is located on the beta-strand of the FAT domain. It has been demonstrated that the variant has a damaging effect on the protein structure and function [47]. Additionally, our results indicated that individuals in a homozygous state for this variant experience bilateral breast cancer at early ages. Furthermore, this variant was found to be very rare in our cohort, with only one case in a heterozygous state out of 400 individuals. Therefore, we have classified the variant as pathogenic.

**Fig. (3):**
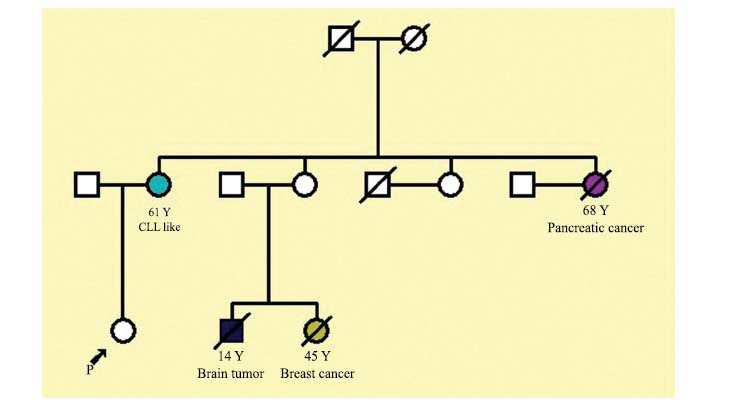
Pedigree of a family with several different cancer diseases (case number three). The proband and her mother are carriers of the *ATM* pathogenic variant (c.7788G>A). The mother, who was detected as a carrier, has developed a CLL-like disease. In this expanded family, several members have passed away due to brain tumors, breast cancer, or pancreatic cancer.

**Fig. (4):**
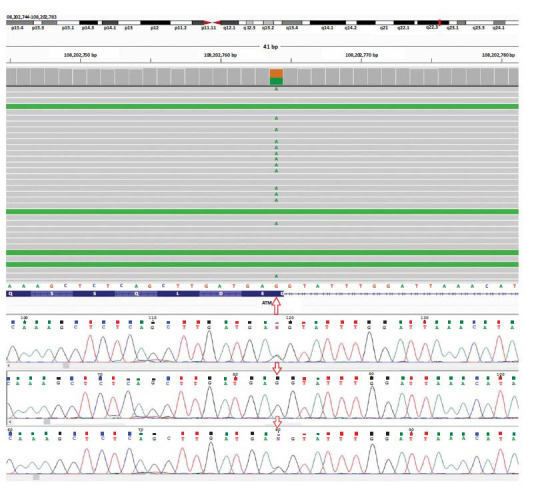
WES results of the third proband (upper figure) show the heterozygous pathogenic variant c.7788G>A at the boundary of exon-intron number 52. The sanger sequencing results of the proband (upper graph) and her parents (father: middle graph, mother; lower graph) confirm the findings of the WES results.

The third case was a forty-year-old woman with a history of different types of cancer in her extended family (Fig. **3**). Most of the affected individuals in this family had developed cancer at later ages of their lives, though two of them developed it at early ages. Her mother developed a CLL-like disease at sixty. Her mother’s sister died at sixty-nine due to pancreatic cancer. Two of the other affected relatives, who were siblings, died due to breast cancer and brain tumor at an early age. Our case has developed no symptoms so far. WES analysis identified the following variant in the heterozygous state: c.7788G>A (p.Glu2596=). This variant is classified as a pathogenic variant as it is located in the boundary of exon-intron number 52 of the *ATM* gene, disrupting the splicing site (splice donor site) (Fig. **4**). Human-splice-finding tool (HSF) confirmed that this pathogenic variant decreased the possibility of identifying this site as a donor site by 88 percent (Delta -88.6%). The score reference was 7.63, whereas this score decreased to 0.87 after the replacement of G with A in the position of 7788 (c.7788G>A). This variant was confirmed by performing Sanger sequencing on both the case and her mother. The trio study revealed that the father was not a carrier for the mutation. In the studied family, it is postulated that most of the carriers of the variant are predisposed to cancer. Furthermore, within this family, three individuals have succumbed to brain tumor, breast cancer, and pancreatic cancer, respectively. Additionally, one individual has been diagnosed with a condition akin to Chronic Lymphocytic Leukemia (CLL) (Fig. **3**). 

To study the frequencies of different variants in the *ATM* gene in our cohort, four hundred individuals were analyzed using the WES technique. Thirty-eight different variants of the *ATM* gene were identified in this cohort (Table **1**). Bioinformatics analysis of the variants showed that all but one of the variants are benign or likely benign. The rs1800057 (*ATM*:c.3161C>G, p.P1054R) variant with a frequency of 8.5% in our cohort was observed in two individuals with a homozygous state and in thirty individuals with a heterozygous state.

## 4 DISCUSSION

Here, we report three families with different pathogenic variants in the *ATM* gene. These variants are c.2639-2A>T, c.8708delC, c.6067G>A, and c.7788G>A. Both of the variants identified in the AT patient, c2639-2A>T and c.8708delC, are novel potentially pathogenic variants. The first variant, c2639-2A>T, located in the boundary region of intron seventeen and exon eighteen, disrupts the splicing procedure by 100 percent. Identification of this variant in a compound heterozygous state in an AT patient and bioinformatics analysis by a Human-splice-finding tool confirmed that this variant is most likely pathogenic. The second novel variant, c.8708delC, removing nucleotide C at position 8708, is located in exon sixty. This variant has a profound effect on the gene product as it causes a frameshift and, most likely, results in the activation of the NMD surveillance pathway and degradation of the mRNA. Our case inherited this variant from his father, and his grandmother (Father’s mother) deceased in her thirties due to a brain tumor. Most likely, she was a carrier for the pathogenic variant, though we had no access to the patient for further genetic analysis. Therefore, we predict that carriers of this variant are severely predisposed to different forms of cancers. None of these variants was observed in 400 individuals comprising our cohort. In our case of AT disease, both variants had a profound effect on the early onset and development of the disease, indicating a significant disruption in the protein functions. It has already been postulated that mutations affecting the splicing process have more severe phenotypes compared to those resulting in missense mutations [45].

The other variant, c.6067G>A, was reported previously in a patient with breast cancer in a compound heterozygous state [46, 47]; however, there was no report of any patient in a homozygous state with this variant. In addition, there have been conflicting interpretations of the pathogenicity of this variant in ClinVar [48]. Yu *et al.* reported a decreased expression of the *ATM* gene by half in immortalized mouse kidney cells (MK4) harboring c.6067G>A variant in a heterozygous state [49]. In this study, we identified the variant in homozygous state in a thirty-eight-year-old woman with bi-lateral breast cancer. No other pathogenic variant related to cancer was identified in this patient by applying WES analysis. Since she had developed bi-lateral breast cancer at an early age, and the variant was not observed in any healthy individual from our cohort, we classified this variant as a pathogenic variant. In addition, bioinformatics analysis confirmed the pathogenicity of the variant.

The c.7788G>A variant (p.Glu2596=), which is located in exon 52 and does not result in any amino acid alteration in the protein, disrupts the splicing procedure by 88 percent (Delta -88.6%) based on results obtained from *in silico* splice site analysis tool, HSF. We identified this variant in a family with different types of cancer. One of the carriers of the pathogenic variant, the mother of our case, developed CLL-like disease at age sixty-nine. Several other members of the family deceased due to pancreatic cancer, breast cancer or brain tumor. Our case, who is a carrier for the variant, has also developed benign cysts in her breasts at age forty. All these data indicate that carriers of this variant are highly predisposed to cancer at different ages, and the penetrance of this variant is quite high.

We continued our study to find all variants of the *ATM* gene and their frequencies in 400 individuals randomly selected from our cohort. Thirty-eight different variants were identified in this cohort, and all but one was benign or likely benign. The only variant that bioinformatics analysis classified as a VUS variant was rs1800057 (P1054R). Meyer *et al.* reported that carriers of this missense variant have an about two-fold increased risk for developing prostate cancer [50]. The frequency of this variant was reported to be 0.009 in the 1000 Genomes Project and 0.016 in the gnomAD project. However, in our cohort, the allele frequency was found to be 0.0425, and the carrier frequency was 0.08. In addition, one individual from our cohort was found to be homozygous for the variant. Observing the higher frequency of the P1054R variant in the Iranian-Azeri Turkish population indicates the possibility of a higher risk of developing prostate cancer or other cancers in this population, which should be under consideration.

## CONCLUSION

In conclusion, we identified two novel pathogenic variants in one patient with AT disease and two other pathogenic variants in the *ATM* gene in two families with cancer from the Iranian Azari Turkish population. Population study revealed rs1800057 variant with relatively higher frequency in this population.

## AUTHORS' CONTRIBUTIONS

ARDA analyzed WES data from the first case. He also designed primers for this family. NJ analyzed WES data from the other two families, designed primers, and analyzed the Sanger sequence. ARDA performed bioinformatics analysis and was a contributor to the preparation of the manuscript. SS performed clinical examinations on patients and contributed to the preparation of the manuscript. MB designed the project, contributed to analyzing the WES results and was a main contributor in manuscript preparation. All authors have read and approved the manuscript. ARDA and NJ have contributed equally to this work and shared the first authorship.

## Figures and Tables

**Table 1:** Study of *ATM* variants revealed 43 different variants in Iranian Azeri Turkish population. * This variant is the only variant classified as VUS and the rest of the identified variants are benign or likely benign. NA: Not Available. Chr-Pos: Chromosome – Position.

**Chr-pos**	**rs**	**Freq. in Our ** **Cohort**	**1000 Genome**	**Chr-pos**	**rs**	**Freq. in Our ** **Cohort**	**1000 Genome**
11-108119616	rs35813860	0.50925926	NA	11-108175463	rs1801673	0.01851852	0.002
11-108100105	rs11390378	0.21296296	NA	11-108186610	rs11212587	0.01851852	0.001
11-108114662	rs768748099	0.11111111	NA	11-108117787	rs28904919	0.00925926	0.001
11-108119616	rs35813860	0.08333333	NA	11-108138003	rs1800056	0.00925926	0.005
11-108121410	rs34325032	0.30555556	NA	11-108143456	rs1800057 *	0.085	0.009
11-108122035	-	0.00925926	NA	11-108124761	rs4986761	0.00925926	0.004
11-108129657	rs672655	0.63888889	0.476	11-108137775	rs642496	0.02777778	0.682
11-108141956	rs373881770	0.35185185	NA	11-108143572	rs876659667	0.00925926	NA
11-108143182	rs664677	0.55555556	0.653	11-108151688	rs148368017	0.01851852	0.008
11-108150208	rs1799757	0.13888889	NA	11-108124299	rs172927	0.00925926	0.486
11-108151707	rs3218681	0.52777778	NA	11-108180917	rs3092910	0.00925926	0.006
11-108175394	rs3092829	0.02777778	0.013	11-108098576	rs1800054	0.01851852	0.004
11-108175462	rs1801516	0.09259259	0.067	11-108123551	rs2227922	0.00925926	0.003
11-108183167	rs659243	0.92592593	1	11-108175387	rs3092828	0.01851852	0.002
11-108141701	rs651030	0.08333333	0.373	11-108160350	rs1800058	0.00925926	0.006
11-108143445	rs199543313	0.00925926	0.00019	11-108150351	rs763382531	0.00925926	NA
11-108163487	rs1800889	0.00925926	0.015	11-108181014	rs201963507	0.00925926	NA
11-108100106	rs752156866	0.01851852	NA	11-108163382	rs4988008	0.00925926	0.001
11-108121411	rs1458765658	0.14814815	NA	11-108098693	rs1004820938	0.00925926	NA
11-108126874	rs2235008	0.0462963	0.013	11-108121411	rs34325032	0.14814815	NA
11-108122700	rs2235006	0.00925926	0.003	11-108114661	rs768748099	0.03703704	NA
11-108124486	rs4987951	0.03703704	0.015	11-108119615	rs35813860	0.05555556	NA
11-108175463	rs1801673	0.01851852	0.002	-	-	-	-

## Data Availability

All data generated or analyzed during this study are either included in this published article or can be made available upon request.

## LIST OF ABBREVIATIONS

AT: Ataxia Telangiectasia
*ATM*: Ataxia-telangiectasia Mutated
BWA: Burrows-Wheeler Aligner
DSBs: Double-strand Breaks
HSF: Human-splice-finding Tool
MK4: Mouse Kidney Cells
NMD: Nonsense-Mediated mRNA Decay
VUS: Uncertain Significant
WES: Whole Exome Sequencing
